# Serum Cytokine Profile in Relation to the Severity of Coronary Artery Disease

**DOI:** 10.1155/2017/4013685

**Published:** 2017-03-02

**Authors:** Xiaoyan Min, Miao Lu, Su Tu, Xiangming Wang, Chuanwei Zhou, Sen Wang, Sisi Pang, Jin Qian, Yiyue Ge, Yan Guo, Di Xu, Kejiang Cao

**Affiliations:** ^1^Department of Geriatric Cardiology, The First Affiliated Hospital of Nanjing Medical University, Nanjing 210029, China; ^2^Department of Emergency, Wuxi No. 2 People's Hospital Affiliated Nanjing Medical University, Wuxi 214000, China; ^3^Institute of Pathogenic Microbiology, Jiangsu Provincial Center for Disease Prevention and Control, Nanjing 210009, China; ^4^Department of Cardiology, The First Affiliated Hospital of Nanjing Medical University, Nanjing 210029, China

## Abstract

*Objectives*. To investigate the potential association of a set of serum cytokines with the severity of coronary artery disease (CAD).* Methods*. A total of 201 patients who underwent coronary angiography for chest discomfort were enrolled. The concentrations of serum IFN-*γ*, TNF-*α*, IL-2, IL-4, IL-6, IL-10, IL-9, and IL-17 were determined by xMAP multiplex technology. The CAD severity was assessed by Gensini score (GS).* Results*. The serum levels of TNF-*α*, IL-6, IL-9, IL-10, and IL-17 were significantly higher in high GS group (GS ≥ 38.5) than those in low GS group (GS < 38.5). Positive correlations were also found between these cytokines and the severity of CAD. After adjustment for other associated factors, three serum cytokines (IL-6, IL-9, and IL-17) and two clinical risk factors (creatinine and LDL-C) were identified as the independent predictors of increased severity of CAD. ROC curve analysis revealed that the logistic regression risk prediction model had a good performance on predicting CAD severity.* Conclusions*. Combinatorial analysis of serum cytokines (IL-6, IL-9, and IL-17) with clinical risk factors (creatinine and LDL-C) may contribute to the evaluation of the severity of CAD and may help guide the risk stratification of angina patients, especially in primary health facilities and in the catheter lab resource-limited settings.

## 1. Introduction

Coronary artery disease (CAD) is the most prevalent chronic disease and a leading cause of morbidity and mortality worldwide [1]. The severity of CAD has been proved to be associated with the risk of future cardiovascular events [2, 3]. Hence, understanding its predictors would greatly help in disease prevention and treatment. It is now widely acknowledged that the pathological basis of CAD is atherosclerosis, and inflammation plays a crucial role in atherosclerotic plaque progression, plaque rupture, and thrombosis, which are the initial factors in acute coronary syndrome (ACS) [4, 5]. This recognition has promoted the evaluation of the correlations between inflammatory markers and cardiovascular risk.

Inflammatory markers, such as serum high sensitive C-reactive protein (hs-CRP), inflammatory cytokines, and chemokines, have been implicated in the initiation and progression of CAD. The levels of hs-CRP, a sensitive indicator of inflammation, are significantly higher in ACS patients with unstable plaque and can reflect the prognosis of ACS patients to some extent [6, 7]. Various inflammatory cytokines such as interleukin-2 (IL-2), interleukin-6 (IL-6), interleukin-17 (IL-17), tumor necrosis factor-alpha (TNF-*α*), and interferon-gamma (IFN-*γ*) have been implicated in CAD, and their concentrations have been found to be associated with increased risks in ACS patients [8–13]. Most recently, data have shown that interleukin-9 (IL-9) might mediate inflammatory cell infiltration into atherosclerotic lesions and may also play a role in the atherosclerotic process [14, 15].

However, most previous studies have paid more attention to the relationship between some inflammatory markers and the atherosclerotic progression and instability. Only a few studies have focused on the correlation of inflammatory cytokines and the severity of coronary atherosclerotic plaque burden in CAD patients [16]. The purpose of this study was to evaluate the clinical significance of a panel of serum cytokines (IFN-*γ*, TNF-*α*, IL-2, IL-4, IL-6, IL-10, IL-9, and IL-17) in CAD patients and to investigate their association with the extent and severity of CAD.

## 2. Methods

### 2.1. Study Population and Samples

The patients were selected from individuals with the symptoms of typical or atypical chest discomfort who were admitted to the cardiology unit of our hospital and underwent coronary angiography from Jan. 2014 to Feb. 2015. The diagnostic criterion of CAD was the patients having at least one severe stenosis (*>*50%) in a major coronary artery. The exclusion criteria were established for patients with active inflammatory or infectious disease, malignant tumor, autoimmune disease, coronary artery spasm angina, severe hepatic and renal dysfunction, and valvular heart disease. In addition, patients with recent surgery or injury were also excluded. A total of 201 patients were included in this study, including 45 patients with stable angina pectoris (SAP), 50 patients with unstable angina pectoris (UAP), 54 patients with acute myocardial infarction (AMI), and 52 controls with chest pain but with normal coronary artery angiographies. The investigation was approved by the Ethics Committee of the First Affiliated Hospital of Nanjing Medical University in accordance with the Declaration of Helsinki. Informed consent was obtained from all patients for the use of the serum samples in this study.

The venous blood samples were collected from each patient prior to angiography and aliquots were sent for assessment of biochemical parameters while the remaining samples were processed within 1 h of collection and the serum samples obtained were held at −80°C until used for cytokine analysis.

### 2.2. Demographic and Clinical Data

The clinical characteristics of all patients including age, gender, Body Mass Index (BMI), and previous histories of hypertension, hyperlipidemia, diabetes, and smoking were recorded before coronary angiography. Clinical examination, electrocardiogram, and transthoracic echocardiography were also carried out before angiography. The biochemical parameters were analyzed by the biochemical laboratory of our hospital using standard methods.

### 2.3. Cytokine Assay

The concentrations of the serum cytokines (IFN-*γ*, TNF-*α*, IL-2, IL-4, IL-6, IL-10, IL-9, and IL-17) were determined by xMAP multiplex technology [17] on the Luminex 100 system (Luminex, Austin, TX, USA), using a Human Premixed Multi-Analyte Kit (R&D Systems, Inc., Minneapolis, USA), and further analyzed using Bioplex Manager Software (Bio-Rad Life Sciences, CA, USA). All analyses were carried out in compliance with the manufacturers' instructions. Standard curves were generated for each cytokine, and the mean fluorescence intensity (MFI) of each cytokine in each well was converted into a concentration using the linear portion of the standard.

### 2.4. Coronary Angiography and CAD Severity Determination

Coronary angiography was performed in all patients after admission by two independent experienced cardiologists. The GS system [18] was used to evaluate the extent and severity of CAD. Reductions in coronary lumen diameter of 1–25%, 26–50%, 51–75%, 76–90%, 91–99%, and 100% were evaluated as a score of 1, 2, 4, 8, 16, and 32, respectively. This score was multiplied by a factor accounting for the lesion position of the coronary artery: 5 for the left main coronary artery, 2.5 for the proximal left anterior descending artery or proximal left circumflex artery, 1.5 for the mid-region of the left anterior descending artery, 1 for the distal left anterior descending artery, the mid-distal region of the left circumflex artery or right coronary artery, and 0.5 for other segments. The CAD severity was expressed as the sum of the score for each lesion. According to the median of GS (38.5), all the CAD patients were divided into two groups of low GS group (GS < 38.5) and high GS group (GS ≥ 38.5).

### 2.5. Statistical Analysis

All statistical analyses were performed with SPSS software version 19.0 (IBM SPSS, Inc., Chicago, USA). Quantitative variables were tested for normal distribution by Shapiro-Wilk's test. Normally distributed variables were presented as mean ± standard deviation (SD), while nonnormally distributed variables were presented as median (25th–75th percentile). Categorical variables were expressed as numbers and percentages. One-way ANOVA with post hoc analysis using LSD test or Kruskal-Wallis ANOVA with post hoc analysis using Mann–Whitney* U* test was performed to compare the quantitative variables among the four groups in the study population (control, SAP, UAP, and AMI). Student's *t*-test or Mann–Whitney *U* test was used for the comparison between two groups of quantitative variables. Categorical variables were compared using the chi-square test or Fisher's exact test, as appropriate. Spearman's test was chosen for correlation analysis because the distributions of variables were not normal. Variables with a significance level of *P* < 0.05 in a univariate test were further used in the multivariate logistic regression analysis (stepwise forward) to determine the association of serum cytokines and other risk factors with the severity of CAD. Then a receiver operating characteristic (ROC) curve analysis was applied and area under curve (AUC) was calculated to determine the predictive value of each independent variable for high GS. According to the multivariate logistic regression risk prediction model, the predictive probability for high GS for each patient was calculated by the combination of five independent variables. Then the ROC curve was constructed to evaluate the discriminatory capability of the novel risk prediction model for high GS, and Youden's index was applied to identify the optimal cut-off point. All *P* values were 2-sided, and the results were considered statistically significant if the *P* value was < 0.05.

## 3. Results

### 3.1. Population Characteristics and Serum Cytokine Levels of CAD Patients

As shown in Table 1, the serum FPG levels in all CAD groups were higher than that in control group (*P* < 0.05). The levels of fibrinogen and hs-CRP in AMI patients were higher than those in other three groups (*P* < 0.01) while an opposite result was obtained for the left ventricular ejection fraction (*P* < 0.01). Other clinical factors did not vary significantly in overall groups.

The expression levels of serum TNF-*α* and IL-6 levels were significantly higher in all CAD groups than in control group (*P* < 0.01). The serum levels of IFN-*γ*, IL-9, and IL-17 were higher in patients with ACS than in controls (*P* < 0.01). However, serum IL-4 levels were lower in patients with AMI than in patients with SAP (*P* < 0.05) and in controls (*P* < 0.01). Furthermore, the levels of serum IL-2 and IL-10 did not differ significantly among the four groups (Table 1).

### 3.2. Association between Serum Cytokine Levels and CAD Severity

According to the median of GS, the CAD patients were classified into low GS group (*n* = 73) and high GS group (*n* = 76). The clinical characteristics of patients in low and high GS groups were listed in Table 2. The levels of creatinine (*P* < 0.05), total cholesterol (*P* < 0.05), LDL-C (*P* < 0.01), fibrinogen (*P* < 0.01), and hs-CRP (*P* < 0.01) in high GS group were significantly higher than those in low GS group. We also found that the high GS group had higher percentage of ACS patients as compared with low GS group (*P* < 0.05).

**Figure 1:**
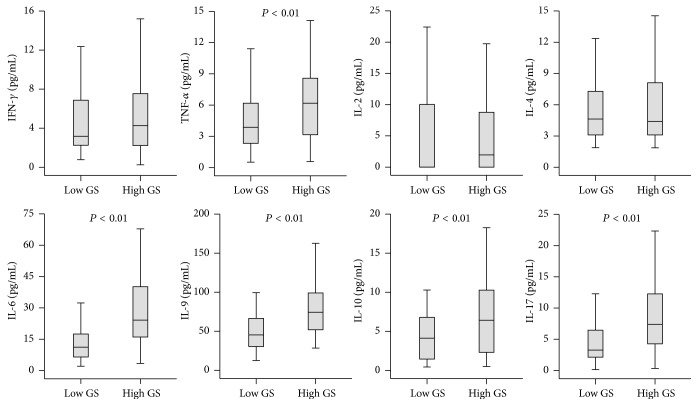
Serum cytokine levels as measured by xMAP multiplex technology in patients with high and low GS. Data are expressed as median with 25th and 75th percentiles.

**Figure 2:**
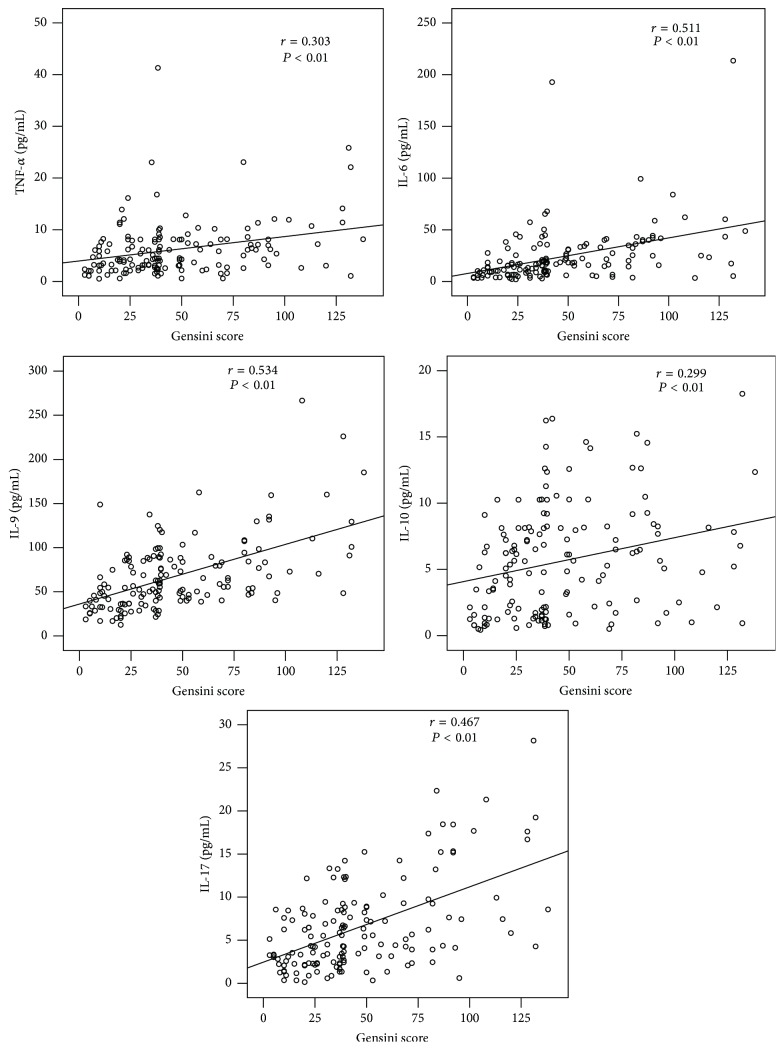
Correlation between serum cytokine (TNF-*α*, IL-6, IL-9, IL-10, and IL-17) levels and GS in CAD patients.* r*, correlation coefficient.

Compared with patients with low GS, those with high GS had higher levels of serum TNF-*α* (*P* < 0.01), IL-6 (*P* < 0.01), IL-9 (*P* < 0.01), IL-10 (*P* < 0.01), and IL-17 (*P* < 0.01), exhibiting a close relationship between these cytokines and the severity of CAD (Figure 1). To further explore the correlations between these cytokines and CAD severity, Spearman correlation analyses were performed. The results revealed that the CAD severity as assessed by GS was significantly and positively correlated with the levels of IL-6 (*r* = 0.511, *P* < 0.01), IL-9 (*r* = 0.534, *P* < 0.01), and IL-17 (*r* = 0.467, *P* < 0.01). The serum levels of TNF-*α* (*r* = 0.303, *P* < 0.01) and IL-10 (*r* = 0.299, *P* < 0.01) were weakly associated with the GS in CAD patients (Figure 2).

### 3.3. Independence of Serum Cytokines in Predicting High GS

As indicated in Table 3, multivariate logistic regression analysis was performed to evaluate the value of serum cytokines in predicting high GS. In this analysis, high GS was employed as a dependent variable, while creatinine, total cholesterol, LDL-C, fibrinogen, hs-CRP, and the serum levels of TNF-*α*, IL-6, IL-9, IL-10, and IL-17 that had a significance level of *P* < 0.05 in univariate tests were set as independent variables. As acute coronary events, especially AMI, can also promote inflammation and upregulation of cytokine production, we also set ACS as an independent variable. After adjustment for other associated factors including ACS, serum levels of IL-6 (OR = 1.043, 95% CI 1.008–1.080, *P* < 0.05), IL-9 (OR = 1.024, 95% CI 1.008–1.039, *P* < 0.01), and IL-17 (OR = 1.189, 95% CI 1.055–1.341, *P* < 0.01) were proved to be independent predictors of high GS. Additionally, our multivariate logistic regression model also confirmed the independence of two clinical risk factors: creatinine (OR = 1.035, 95% CI 1.007–1.063, *P* < 0.05) and LDL-C (OR = 2.190, 95% CI 1.188–4.040, *P* < 0.05) (Table 3).

### 3.4. Utility of Serum Cytokines in Discriminating CAD Severity

**Figure 3:**
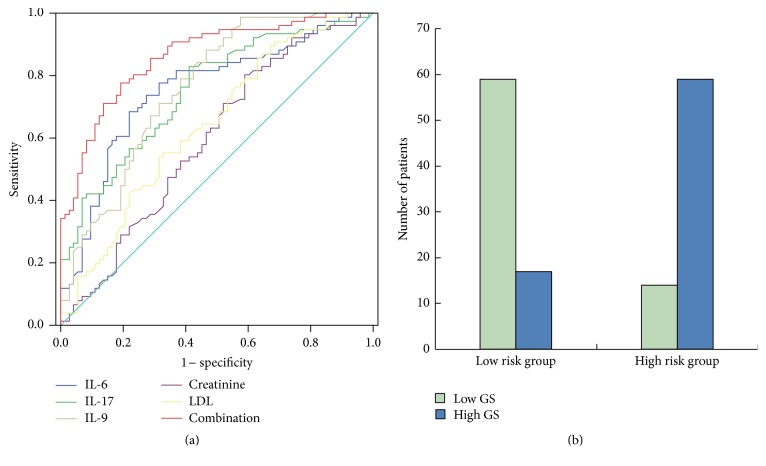
The abilities of serum cytokines in predicting high GS. (a) ROC curves of IL-6, IL-9, IL-17, creatinine, LDL-C, and their combination showing different abilities to predict high GS. (b) Comparison of the distribution of patients between the groups classified according to the optimal cut-off value of the risk prediction model.

ROC curve analysis revealed that the serum levels of IL-6, IL-9, and IL-17 showed moderate abilities in predicting high GS, with an AUC of 0.754 (95% CI: 0.675–0.834, *P* < 0.01), 0.762 (95% CI: 0.686–0.839, *P* < 0.01), and 0.751 (95% CI: 0.673–0.828, *P* < 0.01), respectively (Figure 3(a)). The predictive values of serum creatinine and LDL-C were also indicated in Figure 3(a).

To further evaluate the diagnostic value of the novel logistic regression risk prediction model, the predictive probability of high GS was calculated by the combination of IL-6, IL-9, IL-17, creatinine, and LDL-C for each patient and then subjected to ROC analysis. By combining the five independent factors, the AUC was increased to 0.862 (95% CI: 0.804–0.920, *P* < 0.01, Figure 3(a)). At the optimal cut-off value of 0.47, the sensitivity and specificity reached 77.6% and 80.8%, respectively. Using this cut-off value, all the CAD patients were further divided into two groups (high risk group and low risk group). Compared with low risk group, the high risk group had much more patients with high GS (Figure 3(b)).

## 4. Discussion

CAD remains a health threat worldwide. Identifying the predictors of CAD severity would greatly improve disease prevention, diagnosis, and treatment. In the present study, we demonstrated a strong association between the levels of a panel of serum cytokines and the severity of CAD as assessed by GS. On multivariate analysis, after adjustment for other associated factors, the higher levels of serum IL-6, IL-9, and IL-17 were significantly associated with CAD severity. By combining serum cytokines and clinical cardiovascular risk factors, a novel risk prediction model was constructed. ROC analysis showed that this model had a good performance on the discrimination of high GS, with a sensitivity and specificity of 77.6% and 80.8%, respectively.

Cytokines are the “messengers” of inflammation and immunity which are involved in mediating all stages of atherosclerosis. Various inflammatory cytokines have been implicated in CAD although some of the results were controversial. In our study, the serum levels of IL-6, TNF-*α*, and IFN-*γ* were significantly higher in ACS patients than in controls, which was in accordance with previous studies [8, 14, 19–21]. As an anti-inflammatory cytokine, the serum IL-4 levels in CAD patients were controversial [22–24]. We found lower levels of IL-4 in AMI patients, as compared with controls and SAP patients. IL-10 can reduce the possibility of unstable atherosclerotic plaque formation [25] and have a protective role against plaque rupture [26]. In this study, although no difference of IL-10 levels was found in CAD patients and controls, higher levels of IL-10 were found in patients with high GS. Besides classical Th1/Th2 cytokines, IL-17 was found to promote atherosclerotic lesion development and to be related to the onset of CAD [12, 27]. Increased IL-9 plasma levels were found in patients with ACS or atherosclerosis [15, 28]. Thus, in this study, we also evaluated the serum levels of IL-17 and IL-9 which had not been fully investigated in CAD. The results showed that IL-9 and IL-17 levels were significantly higher in ACS patients than in controls.

As the gold diagnosis standard for CAD, angiography is used not only to confirm the disease but also to evaluatethe extent and severity of atherosclerotic lesions using GS or other scores. GS effectively reflects the severity of coronary stenoses and thus predicts the risk of cardiovascular events [29]. Considering the important role of cytokines in the pathogenesis of CAD, it is plausible that their expression levels may be associated with the disease severity of CAD, even though cytokines are not part of the biomarkers commonly measured in CAD. Indeed, it has been reported that IL-6 and TNF-*α* are significant predictors of the severity of CAD as assessed by GS [16]. Nevertheless, circulating biomarkers have been proven to have limited value in clinical tests to identify severe CAD, primarily because most studies have used single or only a few markers to make the diagnosis. Therefore, in this study, we evaluated a set of serum cytokines which had not been fully investigated and might associate with the disease severity of CAD patients. Our results showed that patients in high GS group had higher levels of serum TNF-*α*, IL-6, IL-9, IL-10, and IL-17 as compared with the low GS group. This suggests that, in addition to IL-6 and TNF-*α* which have been previously showed to associate with the severity of CAD, IL-9, IL-10, and IL-17 might also be predictors of CAD severity. Spearman correlation analyses confirmed that the levels of serum IL-6, IL-9, and IL-17 were significantly associated with the GS of the CAD patients.

To evaluate the value of serum cytokines in predicting high GS, multivariate logistic regression analysis was performed. As ACS patients are likely to be more heterogeneous and inflammatory cytokines are also upregulated during acute coronary events, especially AMI, the findings of the association between serum cytokines and the severity of CAD may be influenced. To account for these possible confounding effects, we set ACS as an independent variable in the logistic regression analysis [30]. After adjustment for other associated factors including ACS, three serum cytokines (IL-6, IL-9, and IL-17) and two clinical risk factors (creatinine and LDL-C) were finally identified as the independent predictors of high GS. Ideal biomarkers are both sensitive and specific to the disease state being examined. However, using a single or only a few biomarkers to identify the complex patient often has a poor specificity [31]. In this study, by the combination of a set of serum cytokines and clinical cardiovascular risk factors, the novel risk prediction model is a more comprehensive indicator for the prediction of high GS. At an optimal cut-off value, the sensitivity and specificity reached 77.6% and 80.8%, respectively.

Although angiography is more precise than our predictive model in evaluating the severity of CAD, our model in combination with other routine clinical tests may provide a low cost, low risk, and clinically useful tool to guide coronary angiography in symptomatic patients, especially in primary health facilities and in the settings where catheter lab resources are limited. For example, patients with mild CAD could be identified by the model without cardiac catheterization thereby reducing their economic burden and also avoiding the exposure to ionizing radiation. For these patients, other noninvasive examinations as well as further follow-up might be considered. Oppositely, for patients likely to be severe CAD, especially in the catheter lab resource-limited settings, transferring them to hospitals with certain facilities for angiographic testing is highly recommended.

## 5. Study Limitations

The following limitations of the study should be considered. Firstly, the number of subjects was relatively small. Secondly, besides the eight serum cytokines investigated in this study, some other cytokines not involved might also associate with the disease severity in CAD patients. Finally, this is a cross-sectional study, with the lack of follow-up data. Therefore, further prospective studies are needed to verify the efficacy of the serum cytokines.

## 6. Conclusions

Serum IL-6, IL-9, and IL-17 are significant predictors of high GS. By combining the levels of serum IL-6, IL-9, and IL-17 with the clinical risk factors creatinine and LDL-C, the risk prediction model constructed in this study may contribute to the evaluation of CAD severity and may provide a clinically useful, low cost, and low risk tool to facilitate the decision-making process for performing angiography in symptomatic patients, especially in primary health facilities and in the catheter lab resource-limited settings.

## Figures and Tables

**Table 1:** Clinical characteristics and serum cytokine levels of patients in each group.

	CAD (*n* = 149)				*P*
	Control (*n* = 52)	SAP (*n* = 45)	UAP (*n* = 50)	AMI (*n* = 54)	
Clinical characteristics					
Age (years)	60.65 ± 9.84	63.16 ± 10.63	64.60 ± 8.81	64.56 ± 14.34	0.237
Male gender, *n* (%)	32 (61.50)	32 (71.10)	34 (68.00)	39 (72.20)	0.650
Hypertension, *n* (%)	25 (48.10)	27 (60.00)	25 (50.00)	29 (53.70)	0.665
Hyperlipidemia, *n* (%)	6 (11.50)	3 (6.70)	5 (10.00)	4 (7.40)	0.816
Diabetes, *n* (%)	14 (26.90)	16 (35.60)	18 (36.00)	15 (27.80)	0.645
Smoking, *n* (%)	11 (21.20)	11 (24.40)	10 (20.00)	7 (13.00)	0.517
BMI (kg/m^2^)	24.63 ± 1.61	24.85 ± 1.71	24.93 ± 1.78	25.12 ± 2.18	0.656
FPG (mmol/L)	5.19 ± 0.94	6.23 ± 1.83^a^	5.92 ± 1.90^a^	6.01 ± 1.97^a^	0.016
Creatinine (*μ*mol/L)	67.92 ± 18.96	69.15 ± 19.56	69.24 ± 16.98	72.77 ± 13.79	0.513
Triglyceride (mmol/L)	1.27 ± 0.57	1.56 ± 0.99	1.27 ± 0.57	1.34 ± 0.98	0.135
Total cholesterol (mmol/L)	4.24 ± 0.95	3.92 ± 1.03	4.24 ± 0.89	4.32 ± 1.00	0.179
HDL-C (mmol/L)	1.12 ± 0.25	1.03 ± 0.25	1.08 ± 0.29	1.03 ± 0.22	0.251
LDL-C (mmol/L)	2.76 ± 0.74	2.49 ± 0.75	2.63 ± 0.73	2.80 ± 0.72	0.153
Fibrinogen (g/L)	2.50 ± 0.48	2.70 ± 0.61	2.68 ± 0.48	3.69 ± 1.18^a,b,c^	<0.01
Hs-CRP (mg/L)	0.93 ± 0.58	1.94 ± 1.56	2.09 ± 1.74	21.16 ± 8.75^a,b,c^	<0.01
Ejection fraction (%)	65.40 (62.55–66.88)	64.40 (62.10–66.40)	65.05 (62.95–67.43)	62.10 (55.90–64.78)^a,b,c^	<0.01
Serum cytokines					
IFN-*γ* (pg/mL)	2.20 (1.44–3.42)	2.68 (1.30–3.37)	3.55 (2.32–4.91)^a,b^	7.54 (3.71–10.17)^a,b,c^	<0.01
TNF-*α* (pg/mL)	2.16 (1.30–4.11)	3.68 (2.24–6.00)^a^	6.00 (2.35–8.12)^a^	6.09 (3.14–8.57)^a,b^	<0.01
IL-2 (pg/mL)	2.55 (0.00–5.28)	1.43 (0.00–8.30)	1.28 (0.00–9.63)	1.95 (0.00–12.26)	0.413
IL-4 (pg/mL)	6.39 (4.28–8.14)	6.36 (3.13–8.25)	5.54 (3.12–8.11)	3.78 (2.68–6.36)^a,B^	<0.01
IL-6 (pg/mL)	6.99 (4.87–11.42)	14.13 (5.37–21.98)^a^	17.17 (9.52–25.16)^a^	22.16 (10.83–40.89)^a,b,C^	<0.01
IL-9 (pg/mL)	30.66 (22.62–42.99)	36.54 (28.00–62.85)	64.65 (47.72–88.55)^a,b^	70.96 (48.56–93.88)^a,b^	<0.01
IL-10 (pg/mL)	4.20 (2.06–5.38)	4.85 (2.16–7.43)	5.49 (1.28–8.46)	5.89 (1.71–8.62)	0.173
IL-17 (pg/mL)	2.65 (2.12–4.57)	3.45 (2.25–6.22)	4.33 (2.52–8.10)^a^	7.65 (3.86–12.23)^a,b,C^	<0.01

Values are expressed as percentages, mean ± SD, or median (25th–75th percentile).

^a^*P* < 0.01 versus control;  ^b^*P* < 0.01 versus SAP;  ^B^*P* < 0.05 versus SAP;  ^c^*P* < 0.01 versus UAP;  ^C^*P* < 0.05 versus UAP.

SAP: stable angina pectoris; UAP: unstable angina pectoris; AMI: acute myocardial infarction; BMI: Body Mass Index; FPG: fasting plasma glucose; HDL-C: high-density lipoprotein cholesterol; LDL-C: low-density lipoprotein cholesterol; Hs-CRP: high sensitive C-reactive protein.

**Table 2:** Clinical characteristics of patients in low and high GS groups.

	Low GS group (GS < 38.5, *n* = 73)	High GS group (GS ≥ 38.5, *n* = 76)	*P*
Age (years)	63.03 ± 10.44	65.22 ± 12.50	0.245
Male gender, *n* (%)	47 (64.40)	58 (76.30)	0.110
Hypertension, *n* (%)	43 (58.90)	38 (50.00)	0.275
Hyperlipidemia, *n* (%)	7 (9.60)	5 (6.60)	0.500
Diabetes, *n* (%)	25 (34.20)	24 (31.60)	0.729
Smoking, *n* (%)	15 (20.50)	13(17.10)	0.591
BMI (kg/m^2^)	25.04 ± 1.95	24.91 ± 1.79	0.687
FPG (mmol/L)	6.14 ± 1.93	5.96 ± 1.87	0.564
Creatinine (*μ*mol/L)	67.34 ± 18.91	73.53 ± 13.82	0.024
Triglyceride (mmol/L)	1.52 ± 0.98	1.51 ± 1.12	0.933
Total cholesterol (mmol/L)	3.99 ± 0.93	4.35 ± 1.00	0.024
HDL-C (mmol/L)	1.03 ± 0.27	1.06 ± 0.24	0.416
LDL-C (mmol/L)	2.47 ± 0.73	2.84 ± 0.70	<0.01
Fibrinogen (g/L)	2.79 ± 0.75	3.30 ± 1.07	<0.01
Hs-CRP (mg/L)	4.99 ± 6.86	12.76 ± 12.27	<0.01
Ejection fraction (%)	64.00 (61.90–66.30)	64.20 (61.65–66.33)	0.976
ACS, *n* (%)	45 (61.6)	59 (77.6)	0.034

Values are expressed as percentages, mean ± SD or median (25th–75th percentile).

BMI: Body Mass Index; FPG: fasting plasma glucose; HDL-C: high-density lipoprotein cholesterol; LDL-C: low-density lipoprotein cholesterol; Hs-CRP: high sensitive C-reactive protein. ACS: acute coronary syndrome.

**Table 3:** Multivariate logistic regression analysis to predict the severity of CAD.

	OR	95% confidence intervals	*P*
Creatinine	1.035	1.007–1.063	0.014
LDL-C	2.190	1.188–4.040	0.012
IL-6	1.043	1.008–1.080	0.017
IL-9	1.024	1.008–1.039	<0.01
IL-17	1.189	1.055–1.341	<0.01

CAD: coronary artery disease; LDL-C: low-density lipoprotein cholesterol.
